# Insights into the Mechanisms of Reuterin against *Staphylococcus aureus* Based on Membrane Damage and Untargeted Metabolomics

**DOI:** 10.3390/foods12234208

**Published:** 2023-11-22

**Authors:** Mao-Cheng Sun, Dian-Dian Li, Yu-Xin Chen, Xiu-Juan Fan, Yu Gao, Haiqing Ye, Tiehua Zhang, Changhui Zhao

**Affiliations:** 1 College of Food Science and Engineering, Changchun University, Changchun 130022, China; msunchina@foxmail.com (M.-C.S.);; 2 College of Food Science and Engineering, Jilin University, Changchun 130062, China

**Keywords:** reuterin, 3-hydroxypropionaldehyde, 3-HPA, food biopreservative, *Staphylococcus aureus*, antibacterial mechanism, membrane damage, untargeted metabolomics

## Abstract

Reuterin is a dynamic small-molecule complex produced through glycerol fermentation by *Limosilactobacillus reuteri* and has potential as a food biopreservative. Despite its broad-spectrum antimicrobial activity, the underlying mechanism of action of reuterin is still elusive. The present paper aimed to explore the antibacterial mechanism of reuterin and its effects on membrane damage and the intracellular metabolome of *S. aureus*. Our results showed that reuterin has a minimum inhibitory concentration of 18.25 mM against *S. aureus*, based on the 3-hydroxypropionaldehyde level. Key indicators such as extracellular electrical conductivity, membrane potential and permeability were significantly increased, while intracellular pH, ATP and DNA were markedly decreased, implying that reuterin causes a disruption to the structure of the cell membrane. The morphological damage to the cells was confirmed by scanning electron microscopy. Subsequent metabolomic analysis identified significant alterations in metabolites primarily involved in lipid, amino acid, carbohydrate metabolism and phosphotransferase system, which is crucial for cell membrane regulation and energy supply. Consequently, these findings indicated that the antibacterial mechanism of reuterin initially targets lipid and amino acid metabolism, leading to cell membrane damage, which subsequently results in energy metabolism disorder and, ultimately, cell death. This paper offers innovative perspectives on the antibacterial mechanism of reuterin, contributing to its potential application as a food preservative.

## 1. Introduction

Natural preservatives have been increasingly favored in recent years due to consumer concerns about the safety of chemical preservatives [1]. They can be derived from a variety of sources such as plants, animals and microorganisms. For example, microorganism-derived compounds such as nisin, pediocin and Micocin^®^ have been commercially applied as food preservatives [2,3]. However, a narrow antimicrobial spectrum and high costs are challenges to the widespread use of these preservatives. It is therefore imperative to develop novel natural preservatives.

Reuterin is a dynamic small-molecule equilibrated complex composed of 3-hydroxypropionaldehyde (3-HPA), its dimer, its hydrate and acrolein, which is produced from glycerol fermentation by *Limosilactobacillus reuteri* [4,5,6]. Reuterin has received much attention due to its multiple biological effects, such as antimicrobial, anti-infective, anti-inflammatory and anticancer action [6]. Notably, reuterin is a promising antimicrobial agent in the food industries because of its potent broad-spectrum antimicrobial activities in various foodstuffs, including meat, dairy products and fresh fruits [6]. Reuterin can inhibit a variety of spoilage organisms and pathogens, such as bacteria, yeast and molds. However, its antibacterial mechanism remains elusive because of the high complexity of reuterin. The action mode of reuterin may be attributed to inhibition of deoxyribonucleic acid (DNA) synthesis in cells by competition (3-HPA dimer) with ribonucleotide reductase, or by reaction (3-HPA or acrolein) with free sulfhydryl groups of certain molecules such as reduced glutathione, thioredoxin or ribonucleotide reductase [4,7,8]. Interestingly, some researchers did not support the above speculation [5,8,9].

*Staphylococcus aureus* is a dangerous foodborne pathogen that can trigger a range of serious and sometime fatal diseases [10]. It can easily contaminate bread, eggs, meat and dairy products [11]. In addition, *S. aureus* easily colonizes surfaces of food and medical equipment, and then forms biofilms that are difficult to remove [12]. Reuterin can effectively inactivate *S. aureus* including its methicillin-resistant strains [13,14]. To our knowledge, there are no reports on the action mode of inhibition of *S. aureus* by reuterin. Therefore, the paper aimed to investigate the potential mechanisms of reuterin against *S. aureus* in the view of membrane damage and intracellular metabolome.

## 2. Materials and Methods

### 2.1. Bacterial Strains

*L. reuteri* ATCC 53608 was obtained from the China Center of Industrial Culture Collection (Beijing, China) and grown in a modified de Man–Rogosa–Sharpe (MRS) broth (Basebio, Hangzhou, China) at 37 °C for the production of reuterin. *S. aureus* ATCC 6538, used as the target microorganism, was purchased from the Shanghai Bioresource Collection Center (Shanghai, China) and grown in tryptic soy broth (TSB) medium (Aoboxing, Beijing, China) at 37 °C.

### 2.2. Production and Quantitation of Reuterin Stock

Reuterin was prepared by a two-step approach [15]. In brief, *L. reuteri* was grown in MRS broth containing 20 mM glycerol at 37 °C for 12 h and then centrifuged (4000× *g*, 10 min, room temperature). The precipitate was washed twice with normal saline and resuspended in 400 mM glycerol/H_2_O solution and then incubated at 37 °C for 1 h. Reuterin stock was obtained by centrifugation of the suspension again, then sterile filtration (0.22 µm), and it was stored at 4 °C until further use. The 3-HPA content was measured using HCl–tryptophan solution via a colorimetric method [16]. The concentration of acrolein was determined using high-performance liquid chromatography (HPLC) with a UV detector (LC-20A, Shimadzu, Kyoto, Japan) as described in [17], and the standard was acrolein-2,4-dinitrophenylhydrazine (Aladdin, Shanghai, China).

### 2.3. Determination of Minimum Inhibitory Concentration (MIC) Value

The MIC (measurement unit: mM) of reuterin, based on 3-HPA, was determined as in the previous research with some modifications [18]. In brief, the cells were diluted to 5 × 10^6^ CFU/mL in TSB media. Subsequently, 100 μL of reuterin stock and 100 μL of prepared bacterial suspension were added to a 96-well cell culture plate and incubated at 37 °C for 16 h. The MIC of reuterin was defined as the lowest concentration that completely inhibited visible growth.

### 2.4. Growth Inhibition Curves

The growth inhibition curves of *S. aureus* under the treatment of reuterin were performed as described previously [19]. In brief, tested bacteria suspensions (5 × 10^6^ CFU/mL) were prepared in a volume of 5 milliliters and kept in test tubes. The final concentration of reuterin was adjusted to 0 (control), 1/2, 1 and 2 MIC. All samples were incubated at 37 °C for 0, 2, 4, 6 and 8 h, respectively. The OD_600nm_ was detected via a SpectraMax M3 microplate reader (Molecular Devices, Sunnyvale, CA, USA).

### 2.5. Extracellular Electrical Conductivity Assay

The electrical conductivity in the supernatant of *S. aureus* was determined following a previous protocol [20]. In brief, the cells during the log phase were washed and resuspended in 0.1M PBS solution. These suspensions were treated with reuterin at final concentrations of 0, 1/2, 1 or 2 MIC. The conductivity was measured every 2 h over an 8-h period using a conductivity meter (ST3100C, OHAUS, Pine Brook, NJ, USA).

### 2.6. Membrane Potential Assay

The membrane potential of *S. aureus* was evaluated according to previous research [21]. In brief, 125 μL of *S. aureus* suspension (OD_600nm_ = 0.5) and 0.5 μL of bis-(1,3-dibutylbarbituric acid) trimethine oxonol (DiBAC4(3)) (AAT Bioquest Inc., Pleasanton, CA, USA) were added to a black opaque 96-well plate and incubated at 37 °C. Then, reuterin was added at final concentrations of 0, 1/2, 1 and 2 MIC. The fluorescence intensity was assessed via a multifunctional microplate reader at excitation and emission wavelengths of 492 and 515 nm, respectively, after incubating for 0.5 h.

### 2.7. Intracellular pH Assay

The impact of reuterin on intracellular pH of *S. aureus* was assessed according to previous research [22]. In brief, the cells at a 5 × 10^8^ CFU/mL concentration were washed twice and resuspended in 50 mM HEPES containing 5 mM EDTA (pH = 8.0). The suspension was then treated with 1.0 μM carboxyfluorescein succinimidyl ester (cFDA-SE) (HΛWN, Shanghai, China) and incubated at 37 °C for 10 min. Bacteria were harvested, suspended in 50 mM PBS and treated with 10 μM glucose before incubating (37 °C, 30 min). The cell pellets were washed twice, resuspended in PBS and kept on ice. The suspension was treated with reuterin at a concentration of 0, 1/2, 1 and 2 MIC at 37 °C for 20 min. The mixed samples were added to a black and opaque 96-well plate. The fluorescence intensities were determined via a multifunctional plate reader at two different excitation wavelengths (440 and 490 nm), and the same emission wavelength of 520 nm, respectively. The intracellular pH was defined as the ratio of fluorescence intensities (490/440 nm emission ratio). The fluorescence values of PBS were detected to remove the background error.

### 2.8. Membrane Permeability Assay

The membrane permeability of *S. aureus* after reuterin treatment was assessed using the method of propidium iodide (PI) staining, as previously reported by Chen et al. [23]. In brief, the cells (10^7^ CFU/mL) were treated with the different levels of reuterin for 4 h. After centrifugation (8000× *g*, 10 min), the collected bacteria were stained with 5 μmol PI (Biosharp, Hefei, China) for 15 min in the dark. The membrane permeability was evaluated by a flow cytometer (BD FACSCalibur, BD Bioscience, San Jose, CA, USA).

### 2.9. Intracellular Adenosine Triphosphate (ATP) Level Assay

The ATP level in *S. aureus* after reuterin treatment was assessed as described previously [24]. In brief, the cells in a logarithmic growth phase were harvested by centrifugation (8000× *g*, 5 min). These bacteria were washed twice and resuspended in PBS. This suspension was incubated at 37 °C for 4 h in the presence of reuterin at concentration 0 (control), 1/2, 1 and 2 MIC, respectively. ATP content in the collected cells was analyzed via an ATP assay kit (Beyotime Biotechnology, Shanghai, China).

### 2.10. Leakage of Intracellular DNA

The intracellular DNA level of *S. aureus* after reuterin treatment was measured with agarose gel electrophoresis, as previously described [20]. In brief, the cells (5 × 10^8^ CFU/mL) were treated with 1 MIC of reuterin and incubated at 37 °C for 4 h. DNA was extracted every 1 h using a bacterial genomic DNA extraction kit (TIANGEN, Beijing, China). The extracted DNA was then measured via electrophoresis with 1.0% agarose gel at 120 V for 0.5 h. The gels were stained by gold view (Yuanye Bio-Tech, Shanghai, China) and visualized by a ZF-288 gel imaging system (JIAPENG, Shanghai, China).

### 2.11. Bacterial Morphology Assay

The cell morphology of *S. aureus* after reuterin treatment was observed under scanning electron microscopy (SEM) according to the procedure used by Kang et al. [25]. In brief, logarithmic phase cells were exposed to reuterin at the different concentrations for 4 h. The cells were washed twice with PBS and fixed in 2.5% glutaraldehyde at 4 °C overnight. All samples were dehydrated in sequential ethanol, lyophilized, coated with gold and, finally, visualized via a JSM-7500F SEM (JEOL, Tokyo, Japan).

### 2.12. LC–MS-Based Untargeted Metabolomic Analysis

Samples were prepared as described previously [26]. In brief, log-phase bacteria were exposed to 0 (control) and 1 MIC of reuterin at 37 °C for 4 h, respectively. These cells were washed 3 times with PBS at 4 °C. After the addition of 400 μL cold methanol (80%), samples were treated in liquid N_2_ for 5 min and melted on ice. Intracellular metabolites were extracted by sonification on an ice water bath for 6 min. The supernatant from sample centrifugation was freeze-dried and redissolved in an equal volume of 10% cold methanol. In addition, blanks and the quality control (QC) samples were prepared to ensure data stability.

All samples were measured via a vanquish UHPLC system coupled to Q Exactive HF/X mass spectrometer (Thermo Fisher Scientific, Bremen, Germany). The detection parameters of LC–MS were set according to the protocol used by Lu et al. [27]. The metabolites were identified using the mzCloud, mzVault and MassList databases. The pathways were annotated from the KEGG database.

### 2.13. Statistical Analysis

Each experiment was repeated at least 3 times. Membrane damage data were expressed as mean ± standard deviation (SD) and examined using analysis of variance (ANOVA) with Duncan’s test in SPSS 26.0 (SPSS Inc., Chicago, IL, USA). The data processing and multivariate analysis from untargeted metabolomics were carried out following a previous protocol [27], including principal component analysis (PCA), partial least squares discriminant analysis (PLS-DA) and metabolite differential analysis. *p* < 0.05 was considered as significant difference.

## 3. Results and Discussion

### 3.1. Antibacterial Activity

The 3-HPA and acrolein concentration in the prepared reuterin stock was 146 mM and 0.0125 mM, respectively, which was similar to the results of Ju et al. [17]. In addition, the MIC value of reuterin against *S. aureus* ATCC 6538 based on 3-HPA level was 18.25 mM, which was different from Ortiz-Rivera et al. [28] and Bennett et al. [29]. The inconsistent results are probably due to the different strains of *S. aureus*.

**Figure 1:**
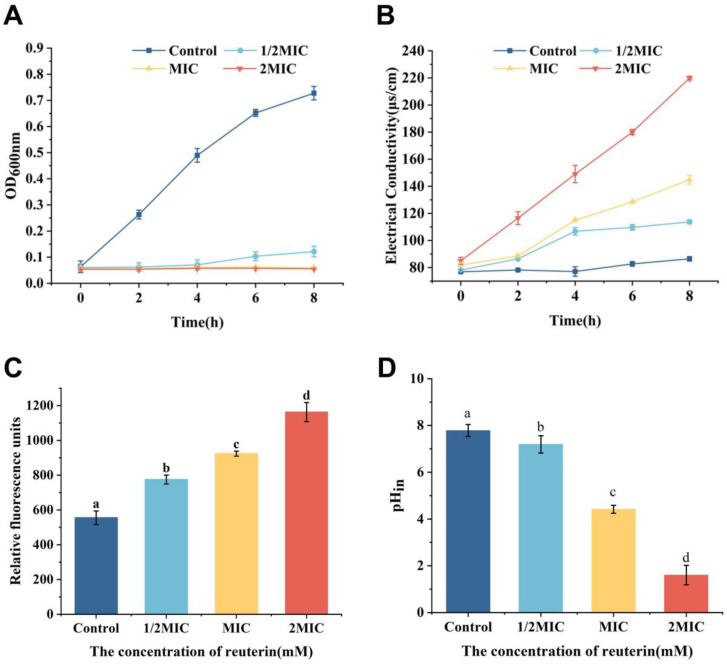
Effect of reuterin on (**A**) growth curves, (**B**) extracellular electrical conductivity, (**C**) membrane potential and (**D**) intracellular pH of *S. aureus* ATCC 6538 (*n* = 3). Letters a–d: different letters on the error bars for each indicator show significant differences (*p* < 0.05).

The growth inhibition curves of reuterin on *S. aureus* are shown in Figure 1A. *S. aureus* treated with reuterin was obviously inhibited compared to the control group (0 MIC), which was similar to Ortiz-Rivera et al. [28]. When treated with 1/2 MIC of reuterin, the strain started to grow slowly only after 4 h. When exposed to 1 and 2 MIC, *S. aureus* was completely inhibited. These data confirmed that reuterin can effectively inhibit the growth of *S. aureus*.

### 3.2. Effect of Reuterin on the Cell Membrane of S. aureus

The bacterial membrane is one of the important targets of many antimicrobial agents [30]. Cell membrane is a phospholipid bilayer composed of phosphatidyl glycerol and cardiolipin, which performs a variety of functions in cell survival such as selective permeability, generation of ATP and cell communication [31]. When the cell membrane is damaged, leakage of intracellular components occurs and the cell morphology is altered. To explore the antibacterial mechanism, we investigated the impacts of reuterin on the cell membrane of *S. aureus* using several methods.

#### 3.2.1. Electrical Conductivity

The conductivity of *S. aureus* suspension can be used to evaluate the release of intracellular small molecules such as potassium and sodium ions, which lead to the change of cell membrane permeability. As is apparent from Figure 1B, the conductivity of the bacterial supernatant significantly increased with increasing reuterin concentration. The values of *S. aureus* treated with 0, 1/2, 1 and 2 MIC of reuterin at 8 h were 86.43 + 1.6, 113.73 + 1.35, 144.90 + 3.31 and 219.8 + 1.40 μs/cm, respectively. The results indicated that reuterin may increase the membrane permeability of *S. aureus* and lead to the efflux of small molecules from cells, thus causing an increase in the conductivity.

#### 3.2.2. Membrane Potential

Membrane potential is a good indicator of membrane damage as it is linked to bacterial replication and ATP synthesis [32]. In this study, DiBAC4(3) was used as a voltage-sensitive fluorescent dye to study the alterations in the membrane potential of *S. aureus*, as it only penetrates the depolarized bacteria and then emits fluorescence by binding to the proteins or membranes [33,34]. As is apparent from Figure 1C, the membrane potential of *S. aureus* after the treatment of reuterin increased in a dose-dependent way and were significantly higher than that of the control group (*p* < 0.05). These data indicated that reuterin caused an increase in depolarization of the cell membrane to raise the membrane potential, which may be due to the disruption of Na^+^/K^+^-ATPase that is essential for maintaining cell membrane function [35].

#### 3.2.3. Intracellular pH of *S. aureus*

In order to further determine the impact of reuterin on the membrane integrity, the changes in intracellular pH were analyzed using the pH-dependent fluorescent dye cFDA-SE. Intracellular pH is strictly maintained in bacteria with an intact membrane and is an effective indicator of cell viability and damage [24]. Figure 1D shows that the intracellular pH of *S. aureus* treated by reuterin decreased significantly with reuterin treatment in a dose-dependent way (*p* < 0.05), compared to the control group. After treating with 1/2, 1 and 2 MIC of reuterin, the intracellular pH values of *S. aureus* decreased by 7.59%, 43.26% and 79.42%, respectively. The results indicated reuterin may cause a decrease in intracellular pH by disrupting the bacterial cell membrane.

#### 3.2.4. PI Staining of *S. aureus*

**Figure 2:**
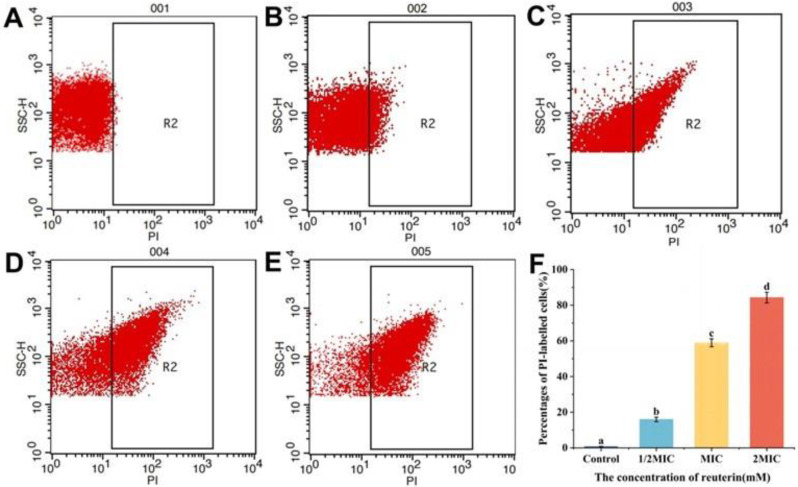
Flow cytometry data of *S. aureus* with PI staining after reuterin treatment. (**A**) Blank cells without PI and reuterin; (**B**) control bacteria without reuterin; (**C**) bacteria exposed to 1/2 MIC reuterin; (**D**) bacteria exposed to 1 MIC reuterin; (**E**) bacteria treated with 2 MIC reuterin; (**F**) the quantitative data (n = 3). Letters a–d: different letters on the error bars show significant differences (*p* < 0.05).

PI dyes only penetrate compromised cell membranes to stain intracellular nucleic acid and then emit red fluorescence [36]. As shown in Figure 2, intact cells appear in the R1 region, while damaged cells appear in the R2 region. The PI-positive bacteria exposed to reuterin at 1/2, 1 and 2 MIC were 14.85%, 58.43% and 81.63%, respectively (Figure 2C–F), while those of untreated cells were only 0.67% (Figure 2B). Compared to the control group, the PI-positive bacteria were raised markedly with the increase in reuterin level (*p* < 0.05, Figure 2F). These results indicated reuterin resulted in the membrane damage in a dose-dependent way.

#### 3.2.5. Intracellular ATP Concentration

**Figure 3:**
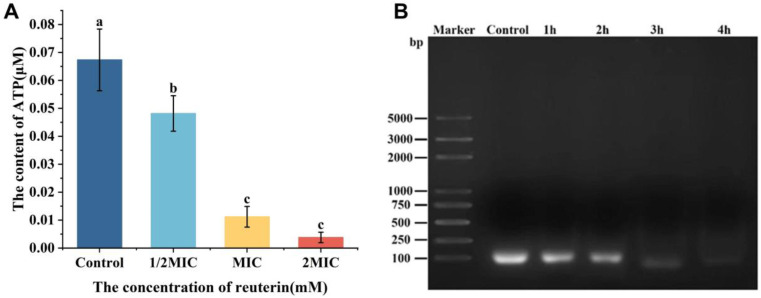
Impact of reuterin on (**A**) intracellular ATP and (**B**) genomic DNA in *S. aureus* (n = 3). Letters a–c: different letters on the error bars show significant differences (*p* < 0.05).

The ATP level in *S. aureus* exposed to reuterin reduced significantly compared to the control (*p* < 0.05; Figure 3A). Furthermore, there was no significant difference between 1 and 2 MIC (*p* > 0.05). The increase in the membrane potential can change membrane permeability, causing the decrease in ATP [37]. The results showed that reuterin could damage the plasma membrane of *S. aureus* to cause the release of intracellular small molecules, resulting in the lower ATP level.

#### 3.2.6. Leakage of the Genomic DNA in *S. aureus*

DNA is essential for bacterial growth, metabolism and reproduction. As is apparent from Figure 3B, the DNA bands of *S. aureus* exposed to reuterin at 1 MIC were obviously fainter with the increase in treatment time compared to the control group. Notably, the DNA band almost disappeared after the 4 h treatment. It indicated that reuterin may cause rapid loss of intracellular DNA or DNA halt.

#### 3.2.7. SEM Analysis

**Figure 4:**
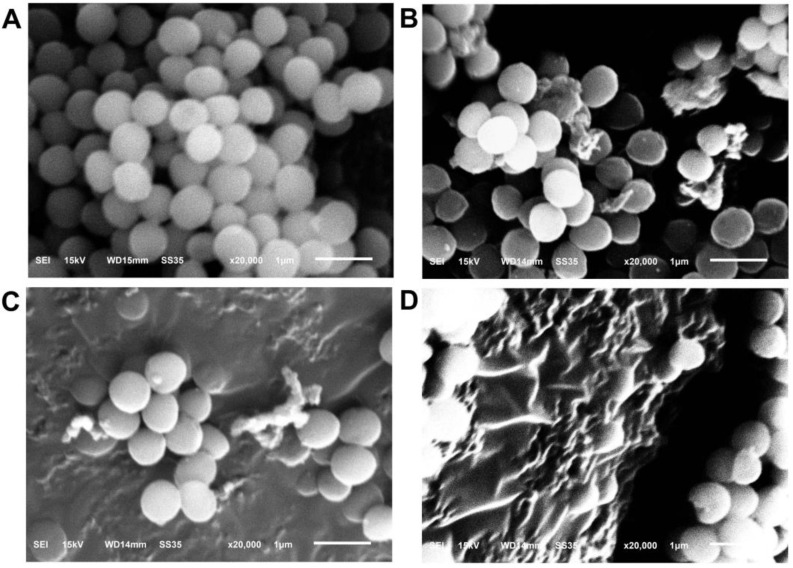
Representative SEM images of *S. aureus* exposed to different levels of reuterin. (**A**) Control; (**B**) 1/2 MIC; (**C**) 1 MIC; (**D**) 2 MIC (n = 3).

The untreated cells featured a regular shape and smooth membrane surface (Figure 4). By treatment with reuterin, more cells were observed to shrink, distort and crack. The treated cells were markedly destroyed with the increase in reuterin content. Therefore, reuterin can destroy the external structures of the cells.

The above assays suggest that reuterin increases the permeability and damages the integrities of the plasma membrane leading to the release of intracellular materials, ultimately causing cell lysis and death. Several previous studies have shown that 3-HPA or acrolein in reuterin reacts with its aldehyde group with SH-containing molecules in bacteria, which induces oxidative damage leading to cell death [4,5]. Cinnamaldehyde, a legally permitted food preservative, is a molecule with a phenyl group attached to an acrolein and can therefore be considered an acrolein derivative [38]. Shen et al. [30] found that the broad-spectrum antibacterial properties of cinnamaldehyde were achieved by targeting the plasma membrane, which may also support the antibacterial mechanism of reuterin in this study. Although, acrolein is more reactive than 3-HPA in terms of chemical structure. The aldehyde group of 3-HPA probably also have some anti-microbial activity. It is speculated that the antibacterial action of reuterin is a result of the synergistic impact from acrolein and 3-HPA.

### 3.3. Untargeted Metabolomic Analysis of S. aureus Treated with Reuterin

**Figure 5:**
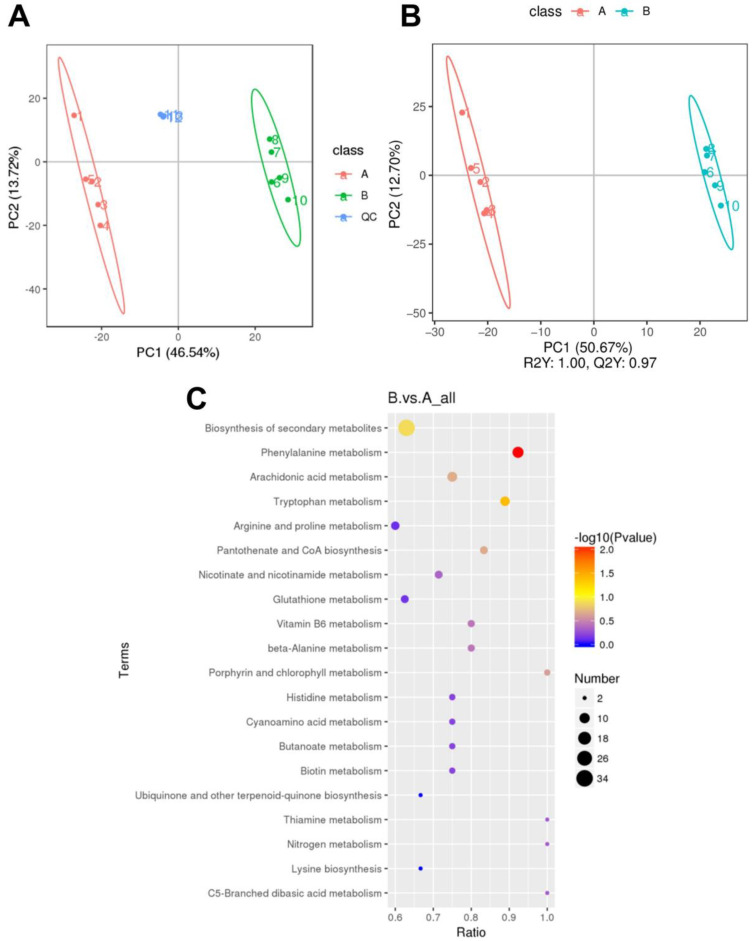
(**A**) Principal component analysis (PCA), (**B**) partial least squares discriminant analysis (PLS-DA) and (**C**) bubble chart of significant metabolic pathways of metabolites of *S. aureus* with reuterin treatment (n = 5). Note: A, control group; B, reuterin treatment at 1 MIC.

We further explored the intracellular metabolites *S. aureus* challenged with reuterin by LC–MS. PCA and PLS-DA were applied to verify differential metabolic profiles between the treated group and control group. Notably, all QC samples were gathered well in Figure 5A, which indicated that the detection platform had good stability and repeatability. As is apparent from Figure 5A,B, the score plots of PCA and PLS-DA presented that the two different groups were completely separated, suggesting that reuterin led to pronounced changes in the intracellular metabolite profile of *S. aureus* compared with the control group.

Significant differences in the metabolites between the two groups were identified on the basis of variable importance in the projection (VIP > 1), fold change (FC > 1.5 or FC < 0.667) and *p* value (T-test, *p* < 0.05). Among them, 147 were upregulated and 372 were downregulated after reuterin treatment, which were annotated into detailed metabolite pathways using KEGG enrichment analysis. As is apparent from Figure 5C, the majority of the disturbed metabolic pathways were involved in phenylalanine metabolism, tryptophan metabolism, arachidonic acid metabolism, pantothenate and CoA biosynthesis and the biosynthesis of secondary metabolites, which proved that reuterin interfered in the intracellular metabolism of *S. aureus*.

#### 3.3.1. Lipid Metabolism

**Figure 6:**
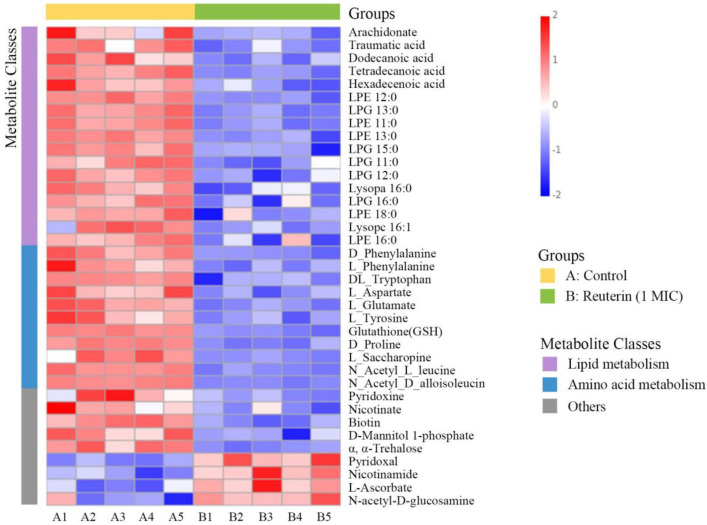
Heatmap of different metabolites in *S. aureus* with or without reuterin at 1 MIC (n = 5). A1 to A5 are the samples from the control group, while B1 to B5 are the samples from the treatment group.

Lipids are important components of microbial membranes and regulates its function to adapt to alterations in the extracellular environment. The structure and composition of lipids affect the flexibility and adaptation capability of cell membranes, which largely determines cell survival [39]. Fatty acid biosynthesis is important for bacteria to alter membrane phospholipids in response to environmental factors [40]. Fatty acid synthesis is a target for antibacterial agent discovery [41]. Additionally, bacterial glycerophospholipid is a constituent of the membrane lipid bilayers which contribute to the selective permeability that permits the transport of essential nutrients or toxins between inside and outside the cell [42]. Metabolomics results showed that fatty acid and glycerophospholipid metabolisms were found to be downregulated after reuterin treatment (Figure 6), indicating that interfering with cell membrane lipid metabolism and causing membrane dysfunction may contribute to the antibacterial mechanism of reuterin.

#### 3.3.2. Amino Acid Metabolism

Many differential metabolites are involved in the inhibition of the amino acid pathway, including phenylalanine metabolism, tryptophan metabolism, beta-alanine, histidine metabolism, cyanoamino acid metabolism, glutathione metabolism, arginine and proline metabolism and lysine biosynthesis. Notably, isoleucine, leucine and its derivatives were downregulated by reuterin treatment (Figure 6). Branched-chain amino acids containing isoleucine and leucine are converted to precursor molecules capable of initiating fatty acid synthesis of *S. aureus*, which contribute to the structure of cell membrane phospholipids [43]. The results indicated that reuterin also damaged the membrane structure, possibly by the disruption of amino acid metabolism. In addition, amino acids biosynthesis, such as phenylalanine and L-glutamate, plays an important role in bacterial survival during some stress treatments [44]. Similarly, the synthesis of amino acids in methicillin-tolerant *S. aureus* is disturbed, resulting in cell death after treatment with teixobactin [45]. It suggested that the self-protection mechanism of the bacterium was disrupted after 1 MIC reuterin treatment.

#### 3.3.3. Other Metabolic Pathways

Vitamin metabolism and carbohydrates metabolism were disturbed after reuterin treatment (Figure 6). Vitamins are vital nutrients for bacteria to grow and metabolize. Similarly, the antibacterial mechanism of lipoic acid also involves membrane damage and imbalance of vitamin metabolism [46]. Notably, the phosphotransferase system (PTS) is essential for the catalysis, uptake, phosphorylation and transport of carbohydrates in bacteria. Imbalance of PTS can lead to disruption of carbohydrate metabolism, which destroys energy metabolism and ultimately leads to bacterial death [47].

**Figure 7:**
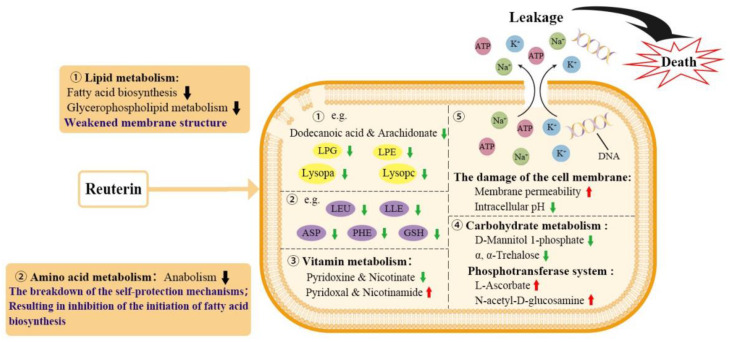
Proposed schematic of antibacterial mechanism of reuterin against *S. aureus*. Red and green arrows indicate up-regulation and down-regulation, respectively.

Therefore, we propose that reuterin first inhibits lipid metabolism and amino acid metabolism, leading to cell membrane damage, and then destroys energy metabolism via PTS, which ultimately leads to death of *S. aureus* (Figure 7).

## 4. Conclusions

This study represents the first investigation into the antimicrobial mechanism of reuterin against *Staphylococcus aureus* using membrane damage assays and untargeted metabolomics. Our findings demonstrated that reuterin can disrupt the cell membrane of *S. aureus* through various mechanisms, including increased extracellular electrical conductivity and dissipation of membrane potential, decreased intracellular pH, enhanced membrane permeability, leakage of intracellular material and damaged morphology. Moreover, metabolomics analysis revealed that reuterin induced significant alterations in many metabolic pathways related to lipid metabolism, amino acid metabolism, PTS and carbohydrate metabolism. Therefore, it appears that the antibacterial mechanism of reuterin initially disrupts the cell membrane by interfering with lipid and amino acid metabolism, subsequently inhibiting carbohydrate metabolism via the PTS, ultimately leading to cell death. However, the identification of specific molecules within the cell membrane targeted by reuterin remains an area for future investigation. The insights gained from this study enhance the comprehension of the anti-*S. aureus* mechanisms of reuterin, providing a theoretical foundation for potential applications of reuterin as food preservatives or health promoters.

## Data Availability

The data used to support the findings of this study can be made available by the corresponding author upon request.

## References

[1] Olatunde O.O., Benjakul S. (2018). Natural preservatives for extending the shelf-life of seafood: A revisit. Compr. Rev. Food Sci. Food Saf..

[2] Barbosa A.A.T., Mantovani H.C., Jain S. (2017). Bacteriocins from lactic acid bacteria and their potential in the preservation of fruit products. Crit. Rev. Biotechnol..

[3] Liu Y. (2014). Heat and Pressure Resistance of *Escherichia coli* and Its Inactivation in the Presence of Antimicrobial Compounds. Ph.D. Thesis.

[4] Engels C., Schwab C., Zhang J., Stevens M.J., Bieri C., Ebert M.O., Mcneill K., Sturla S.J., Lacroix C. (2016). Acrolein contributes strongly to antimicrobial and heterocyclic amine transformation activities of reuterin. Sci. Rep..

[5] Schaefer L., Auchtung T.A., Hermans K.E., Whitehead D., Borhan B., Britton R.A. (2010). The antimicrobial compound reuterin (3-hydroxypropionaldehyde) induces oxidative stress via interaction with thiol groups. Microbiology.

[6] Sun M.C., Hu Z.Y., Li D.D., Chen Y.X., Xi J.H., Zhao C.H. (2022). Application of the Reuterin System as Food Preservative or Health-Promoting Agent: A Critical Review. Foods.

[7] Vollenweider S., Grassi G., Iwo König A., Puhan Z. (2003). Purification and Structural Characterization of 3-Hydroxypropionaldehyde and Its Derivatives. J. Agric. Food Chem..

[8] Vollenweider S., Evers S., Zurbriggen K., Lacroix C. (2010). Unraveling the hydroxypropionaldehyde (HPA) system: An active antimicrobial agent against human pathogens. J. Agric. Food Chem..

[9] Cleusix V., Lacroix C., Vollenweider S., Duboux M., Le Blay G. (2007). Inhibitory activity spectrum of reuterin produced by *Lactobacillus reuteri* against intestinal bacteria. BMC Microbiol..

[10] Piewngam P., Khongthong S., Roekngam N., Theapparat Y., Sunpaweravong S., Faroongsarng D., Otto M. (2023). Probiotic for pathogen-specific *Staphylococcus aureus* decolonisation in Thailand: A phase 2, double-blind, randomised, placebo-controlled trial. Lancet Microbe.

[11] Pal M., Berhanu G., Megersa L., Kandi V. (2020). Epidemiology, Pathogenicity, Animal Infections, Antibiotic Resistance, Public Health Significance, and Economic Impact of *Staphylococcus aureus*: A Comprehensive Review. Am. J. Public Health Res..

[12] Moormeier D.E., Bayles K.W. (2017). *Staphylococcus aureus* biofilm: A complex developmental organism. Mol. Microbiol..

[13] Yehia H.M., Alkhuriji A.F., Savvaidis I., Al-Masoud A.H. (2022). Bactericidal effect of nisin and reuterin on methicillin-resistant *Staphylococcus aureus* (MRSA) and *S. aureus* ATCC 25937. Food Sci. Technol..

[14] Arqués J.L., Rodríguez E., Nuñez M., Medina M. (2011). Combined effect of reuterin and lactic acid bacteria bacteriocins on the inactivation of food-borne pathogens in milk. Food Control.

[15] Vimont A., Fernandez B., Ahmed G., Fortin H.P., Fliss I. (2019). Quantitative antifungal activity of reuterin against food isolates of yeasts and moulds and its potential application in yogurt. Int. J. Food Microbiol..

[16] Circle S.J., Stone L., Boruff C.S. (1945). Acrolein Determination by Means of Tryptophane. A Colorimetric Micromethod. Ind. Eng. Chem..

[17] Ju J.-H., Jeon S.-G., Lee K.M., Heo S.-Y., Kim M.-S., Kim C.-H., Oh B.-R. (2021). The biocatalytic production of 3-hydroxypropionaldehyde and evaluation of its stability. Catalysts.

[18] Urrutia-Baca V.H., Escamilla-Garcia E., de la Garza-Ramos M.A., Tamez-Guerra P., Gomez-Flores R., Urbina-Rios C.S. (2018). In Vitro Antimicrobial Activity and Downregulation of Virulence Gene Expression on *Helicobacter pylori* by Reuterin. Probiotics Antimicrob. Proteins.

[19] Shi C., Zhao X., Meng R., Liu Z., Zhang G., Guo N. (2017). Synergistic antimicrobial effects of nisin and p-Anisaldehyde on *Staphylococcus aureus* in pasteurized milk. LWT.

[20] Tao Y., Qian L.-H., Xie J. (2011). Effect of chitosan on membrane permeability and cell morphology of *Pseudomonas aeruginosa* and *Staphyloccocus aureus*. Carbohydr. Polym..

[21] Sanchez E., Garcia S., Heredia N. (2010). Extracts of edible and medicinal plants damage membranes of Vibrio cholerae. Appl. Env. Microbiol..

[22] Chang Y., Xing M., Hu X., Feng H., Wang Y., Guo B., Sun M., Ma L., Fei P. (2020). Antibacterial Activity of Chrysanthemum buds Crude Extract against *Cronobacter sakazakii* and Its Application as a Natural Disinfectant. Front. Microbiol..

[23] Chen K., Peng C., Chi F., Yu C., Yang Q., Li Z. (2022). Antibacterial and Antibiofilm Activities of Chlorogenic Acid Against Yersinia enterocolitica. Front. Microbiol..

[24] Shi C., Song K., Zhang X., Sun Y., Sui Y., Chen Y., Jia Z., Sun H., Sun Z., Xia X. (2016). Antimicrobial activity and possible mechanism of action of citral against *Cronobacter sakazakii*. PLoS ONE.

[25] Kang J., Jin W., Wang J., Sun Y., Wu X., Liu L. (2019). Antibacterial and anti-biofilm activities of peppermint essential oil against *Staphylococcus aureus*. LWT.

[26] Yuan M., Breitkopf S.B., Yang X., Asara J.M. (2012). A positive/negative ion-switching, targeted mass spectrometry-based metabolomics platform for bodily fluids, cells, and fresh and fixed tissue. Nat. Protoc..

[27] Lu J., Zhang X., Liu Y., Cao H., Han Q., Xie B., Fan L., Li X., Hu J., Yang G. (2019). Effect of fermented corn-soybean meal on serum immunity, the expression of genes related to gut immunity, gut microbiota, and bacterial metabolites in grower-finisher pigs. Front. Microbiol..

[28] Ortiz-Rivera Y., Sanchez-Vega R., Gutierrez-Mendez N., Leon-Felix J., Acosta-Muniz C., Sepulveda D.R. (2017). Production of reuterin in a fermented milk product by *Lactobacillus reuteri*: Inhibition of pathogens, spoilage microorganisms, and lactic acid bacteria. J. Dairy Sci..

[29] Bennett S., Ben Said L., Lacasse P., Malouin F., Fliss I. (2021). Susceptibility to nisin, bactofencin, pediocin and reuterin of multidrug resistant *Staphylococcus aureus*, *Streptococcus dysgalactiae* and *Streptococcus uberis* causing bovine mastitis. Antibiotics.

[30] Shen S., Zhang T., Yuan Y., Lin S., Xu J., Ye H. (2015). Effects of cinnamaldehyde on *Escherichia coli* and *Staphylococcus aureus* membrane. Food Control.

[31] Nikolic P., Mudgil P. (2023). The Cell Wall, Cell Membrane and Virulence Factors of *Staphylococcus aureus* and Their Role in Antibiotic Resistance. Microorganisms.

[32] Yasir M., Dutta D., Willcox M.D.P. (2019). Mode of action of the antimicrobial peptide Mel4 is independent of *Staphylococcus aureus* cell membrane permeability. PLoS ONE.

[33] Clementi E.A., Marks L.R., Roche-Håkansson H., Håkansson A.P. (2014). Monitoring changes in membrane polarity, membrane integrity, and intracellular ion concentrations in *Streptococcus pneumoniae* using fluorescent dyes. J. Vis. Exp..

[34] Morici P., Rizzato C., Ghelardi E., Rossolini G.M., Lupetti A. (2023). Sensitization of KPC and NDM Klebsiella pneumoniae To Rifampicin by the Human Lactoferrin-Derived Peptide hLF1-11. Microbiol. Spectr..

[35] Kumari J., Rathore M.S. (2020). Na+/K+-ATPase a Primary Membrane Transporter: An Overview and Recent Advances with Special Reference to Algae. J. Membr. Biol..

[36] Rosenberg M., Azevedo N.F., Ivask A. (2019). Propidium iodide staining underestimates viability of adherent bacterial cells. Sci. Rep..

[37] Suh S.-J., Shuman J., Carroll L.P., Silo-Suh L. (2016). BEEP: An assay to detect bio-energetic and envelope permeability alterations in *Pseudomonas aeruginosa*. J. Microbiol. Methods.

[38] Muhoza B., Qi B., Harindintwali J.D., Koko M.Y.F., Zhang S., Li Y. (2023). Encapsulation of cinnamaldehyde: An insight on delivery systems and food applications. Crit. Rev. Food Sci. Nutr..

[39] Šajbidor J. (1997). Effect of Some Environmental Factors on the Content and Composition of Microbial Membrane Lipids. Crit. Rev. Biotechnol..

[40] Zhang Y.-M., Rock C.O. (2008). Membrane lipid homeostasis in bacteria. Nat. Rev. Microbiol..

[41] Yao J., Rock C.O. (2017). Bacterial fatty acid metabolism in modern antibiotic discovery. Biochim. Biophys. Acta (BBA)-Mol. Cell Biol. Lipids.

[42] Kamischke C., Fan J., Bergeron J., Kulasekara H.D., Dalebroux Z.D., Burrell A., Kollman J.M., Miller S.I. (2019). The *Acinetobacter baumannii* Mla system and glycerophospholipid transport to the outer membrane. eLife.

[43] Frank M.W., Whaley S.G., Rock C.O. (2021). Branched-chain amino acid metabolism controls membrane phospholipid structure in Staphylococcus aureus. J. Biol. Chem..

[44] Liu M., Feng M., Yang K., Cao Y., Zhang J., Xu J., Hernández S.H., Wei X., Fan M. (2020). Transcriptomic and metabolomic analyses reveal antibacterial mechanism of astringent persimmon tannin against Methicillin-resistant *Staphylococcus aureus* isolated from pork. Food Chem..

[45] Hussein M., Karas J.A., Schneider-Futschik E.K., Chen F., Swarbrick J., Paulin O.K.A., Hoyer D., Baker M., Zhu Y., Li J. (2020). The Killing Mechanism of Teixobactin against Methicillin-Resistant Staphylococcus aureus: An Untargeted Metabolomics Study. mSystems.

[46] Yang S., Tian L., Wang X., Wu M., Liao S., Fu J., Xiong W., Gong G. (2022). Metabolomics analysis and membrane damage measurement reveal the antibacterial mechanism of lipoic acid against Yersinia enterocolitica. Food Funct..

[47] Tan S., Hua X., Xue Z., Ma J. (2020). Cajanin Stilbene Acid Inhibited Vancomycin-Resistant Enterococcus by Inhibiting Phosphotransferase System. Front. Pharmacol..

